# Three Thiol-Reactive Reagents drive Synergistic Lethality in Glioblastoma cells

**DOI:** 10.21203/rs.3.rs-9978519/v1

**Published:** 2026-07-15

**Authors:** Ofer Y. Kashi, Adi Cohen, Kathleen Earhart, Jonathan David Elliot, Denise M. O. Ramirez, Sukanya Rauniyar, Ghazi Qureshi, Gali Umschweif, Evan K. Noch, Daphne Atlas

**Affiliations:** 1 Dept. of Biological Chemistry, Institute of Life Sciences, Edmond J Safra Campus Hebrew University of Jerusalem; 2 The Institute for Drug Research, School of Pharmacy, Faculty of Medicine, The Hebrew University of Jerusalem; 3 Department of Neurology, University of Texas Southwestern Medical Center, Dallas, TX, USA; 4 Peter O’Donnell Jr. Brain Institute, University of Texas Southwestern Medical Center, Dallas, TX, USA

**Keywords:** Glioblastoma, MAPK, STAT3, thioredoxin, acrylamide, BSO, oxidative stress, Auranofin

## Abstract

Glioblastoma (GBM) is the most prevalent malignant primary brain tumor. Disruption of the redox state of the cell through cysteine (Cys) reactive residues has been suggested to play a role in the progression of GBM. Here, we demonstrate that the addition of acrylamide (ACR), a thiol-reactive molecule that covalently modifies redox-related proteins and disrupts critical redox signaling pathways, further amplifies the cytotoxic effects and the synergistic lethality induced by auranofin (Auf) and buthionine sulfoximine (DL-BSO). We established a minimal triple-thiol cocktail of ACR/Auf/BSO, which enhances suppression of cell growth in various cancer cell lines. This combination of ACR, BSO, and Auf, which causes a complete cell death in U87MGMG, U87MGΔEGFR, A431, PC12 and SH-SY5Y cells induces significantly less toxicity in primary rat cortex neuronal cultures. The synergy observed is strongly associated with increased activation of ERK1/2 and p38MAPK phosphorylation, as well as the inhibition of the STAT3 signaling pathways, which are both critical for cell survival and proliferation. In contrast, primary neuronal cortical cells, which exhibit minimal toxicity with the minimal ACR/Auf/BSO combination, display no activation of these antioxidant/anti-inflammatory molecular pathways. The combination of the oxidizing reagents induces whole-cell and mitochondrial reactive oxygen species (ROS) production in patient-derived GBM cells and reduces proliferation of a human GBM organoid model. We suggest that combining multiple Cys-associated redox targets is a putative therapeutic strategy for GBM, which offers a promising approach for improving treatment outcomes in GBM and other malignancies.

## Graphical Abstract

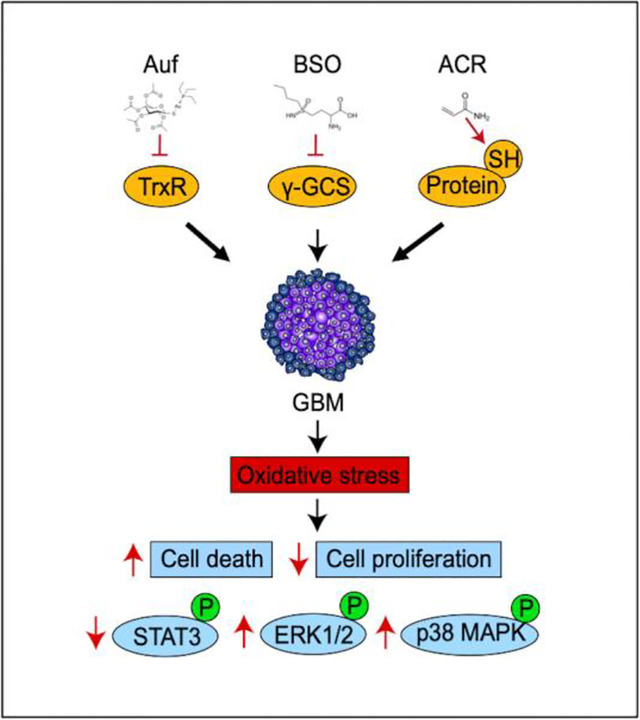

## 1. Introduction

Glioblastoma (GBM) is a highly aggressive and fatal brain cancer. Its progression is influenced by the upregulation of oncogene-controlled membrane transporters and metabolic enzymes, which play a pivotal role in generating NADPH. Sustaining the activity of major antioxidant systems, including the thioredoxin reductase-thioredoxin (TrxR-Trx1) system and the glutathione (GSH)-GSSG system, enables tumor cells to combat oxidative stress and support growth and survival (Zhu and Thompson, 2019). The TrxR-Trx1 and the GSH-GSSG redox systems counteract excessive production of reactive oxygen species (ROS) causing cell damage by oxidizing proteins, lipids, DNA, and RNA. An increase in ROS may also lead to drug resistance shown in GBM, therefore, ROS regulation may represent a critical target for the development of antitumor agents (Nogueira and Hay, 2013), (Perillo et al., 2020), (Kuljanin et al., 2021). GBM is associated with GBM-specific EGFR variants, which function in three major core signaling pathways: G1-S cell cycle checkpoint, the TP53 pathway, and receptor tyrosine kinase (RTK) signaling via downstream mitogen activated protein (MAPK) and phosphatidylinositol 3-kinases (Hanahan, 2022).

Poor prognosis and chemoresistance in GBM, as well as in many other cancers, are strongly associated with overexpression of thioredoxin reductase 1 (TrxR1), making it an attractive therapeutic target (Gencheva and Arner, 2022). Notably, the FDA-approved organo-gold compound auranofin (Auf), originally developed for the treatment of rheumatoid arthritis, is a highly selective TrxR1 inhibitor and has been proposed as an anticancer treatment (Mirabelli et al., 1985).

In recent years Auf was upgraded as an innovative cancer drug for overcoming drug resistance and increasing drug effectiveness (Gamberi et al., 2021). Auf has been demonstrated to promote cell lethality in several cancer types, including high-grade ovarian cancer cells (Abdalbari et al., 2023), acute myeloid leukemia (Cunningham et al., 2024), lymphoma (Yang et al., 2024) and others (Hatem et al., 2022), (Massai et al., 2022), highlighting its potential as an anti-cancer agent. Analogous to other TrxR inhibitors, Auf also inhibits the signal transducer and activator of transcription (STAT3) signaling pathway which is upregulated in GBM and other cancer cells (Busker et al., 2020). Oxidative stress is also triggered by *S*,*R*-buthionine sulfoximine (BSO), an inhibitor of γ-glutamyl-cysteine synthetase, through depletion of intracellular GSH. BSO is an anticancer agent known for its ability to enhance the cytotoxicity of various chemotherapeutic agents and radiation therapy, exhibiting minor toxicity by itself (Dorr et al., 1986), (Lippitz et al., 1990).

Recent studies have highlighted the potential of combining Auf and BSO to induce synergistic cell lethality. When applied together, this combination has demonstrated enhanced anticancer activity in various malignancies, including head and neck cancer (Simons et al., 2009), (Sobhakumari et al., 2012), (Roh et al., 2017), (Fath et al., 2011), B-cell malignancies (Kiebala et al., 2015), breast and prostate cancer (Li et al., 2015), mesothelioma (You and Park, 2016), acute lymphoblastic leukemia (Haβ et al., 2016), rhabdomyosarcoma (Habermann et al., 2017), cervical cancer (Rashmi et al., 2018), pancreatic cancer (Fazzari et al., 2022), lung cancer (Cui et al., 2022) and GBM stem cells (Jamali et al., 2024). Synergistic cytotoxicity by Auf and BSO has also been demonstrated in GBM cells, both in those with aberrant or overexpressed EGFR and those without EGFR overexpression (Martinez-Jaramillo et al., 2024). Here, we aimed to investigate the impact of acrylamide (ACR) on cell death induced by the combination of Auf and BSO in GBM and other cancer cells. ACR, a reactive thiol reagent, covalently binds thiol groups and induces oxidative stress through interacting with cysteine (Cys) residues of redox-associated proteins, such as Trx1, GSH, and glutaredoxin (LoPachin and DeCaprio, 2005). Additionally, the incorporation of an ACR covalent warhead into the structure of selective tyrosine kinase inhibitor drugs has been shown to enhance their efficacy in treating GBM and other types of cancers (Liu et al., 2013). For example, ibrutinib, a drug used to treat mantle cell lymphoma, covalently binds to Cys481 in the ATP-binding pocket of Bruton’s tyrosine kinase, highlighting the therapeutic potential of targeting Cys in cancer-related kinases (Barf and Kaptein, 2012), or irreversibly modifying Cys residues like the C797S mutant of EGFR (Kuki et al., 2023), reviewed in (Liang et al., 2024). We evaluated the cytotoxic effects of Auf, BSO and ACR alone and in combination in GBM and other cancer cell lines to determine the effect of targeting Cys residues using three distinct thiol-reactive reagents. The combination of ACR with the highly effective thiol modifiers Auf and BSO inhibited ERK and p38MAPK activation and reduced STAT3 signaling in cancer cells, suggesting a potential molecular mechanism in triggering cell death. In addition, the combination induced whole-cell and mitochondrial reactive oxygen species (ROS) in patient-derived GBM cells. In a human GBM tissue organoid model, this 3-drug combination reduced proliferation. By simultaneously disrupting multiple cellular pathways, this drug combination holds promise as a novel therapeutic strategy for improving the treatment of GBM and other types of cancer.

## 2. Materials and Methods

### 2.1 Cell lines cultures

Human GBM U87MG, U87MGΔ(EGFR 2–7) cells, epidermal carcinoma cells (A431), human cervical cancer HeLa cells, and human embryonic kidney (HEK293) cells were cultured in high-glucose DMEM (Sartorius, Israel) supplemented with 10% FBS (Capricorn, Germany) 1.5% L-Alanyl-L-glutamine, 1% Penicillin/Streptomycin, and 1% HEPES buffer 1mM (Sartorius, Israel).

Human neuroblastoma (SH-SY5Y) cells were cultured in DMEM/F12 HAM 1:1 medium (Diagnovum, Germany) supplemented with 10% FBS, 1.5% L-Alanyl-L-glutamine (Sartorius), and 1% Penicillin/Streptomycin; Rat pheochromocytoma (PC12) cells were cultured in high glucose DMEM (Sartorius, Israel), supplemented with 12% FBS), 1.5% L-Alanyl-L-glutamine, 1% Penicillin/Streptomycin (Capricorn, Germany), and 1% HEPES buffer 1M pH7.4.

667 GBM spheroid cell cultures were obtained from Cameron Brennan at Memorial Sloan Kettering Cancer Center (MSKCC). The cells were cultured in 1:1 Neurobasal medium and DMEM/F12 medium supplemented with Glutamax, HEPES, sodium pyruvate, minimal essential amino acids, Pen/Strep, B27 supplement minus vitamin A, heparin (2 μg/ml), and EGF and FGF (20 μg/ml) (all from Life Technologies, Waltham, MA, USA).Cell cultures were maintained at 37 °C under 5% CO_2_.

### 2.2 Primary cortex neuronal culture and treatment

Breeding cages of **C57Bl6 mice** were established for 1–2 nights, and pregnancy was identified by visualization of a plug on the female mouse. Embryos extraction was conducted by C-section, 19 days after conception (E19). Brain dissection was conducted in a sterile biological hood, and brains were kept at RT in 5ml HBSS (HBSS, calcium, magnesium solution (Gibco US). Cortexes of 6–9 embryos of the same litter were disintegrated by pipetting and then moved to a fresh tube with the addition of 2.5mL Trypsin-EDTA, following by incubation for 7 min in a culture incubator. Then, 750μL FBS was added to the mix and gently centrifuged for 5 min x1200 RPM. The supernatant was vacuumed and the cells resuspended in complete growth media consisting of Neurobasal media, 2% B-27 (Gibco), 1% L-glutamine (Thermo Scientific), and 1% penicillin-streptomycin, and was strained with a 0.2μM filter. Cells were seeded at a concentration of 200 ×10^3^/well in a 96-well plate or 500 ×10^3^/well in 24 wells, which were pre-coated with Poly-L-lysine (Sigma, Israel). Media was changed after 24 h to a 1:1 mix of complete and conditioning media. Half of the volume was replaced twice a week. Medium from healthy WT neural cultures from DIV7+ was strained in 0.2 μm filter and kept as conditioning media. In a cell viability assay, primary cells were cultured in their plates for two weeks prior to treatment. All methods were performed in accordance with the relevant guidelines and regulations by Assessment and Accreditation of Laboratory Animal Care (AAALAC) of the HUJI’s Authority for Biological and Biomedical Models (ABBM).

### 2.3 Cell viability and methylene blue assay

Cells were seeded in 96 wells plates for 24 hours followed by incubation with different combinations of ACR, AuF, BSO for 72 hours. U87MG and U87MGΔ(EGFR 2–7) cells were seeded at a density of 6 × 10^3^/well in a 96-well plate or 700 × 10^3^ per well in a 24-well plate (Corning, US); A431 cells at 3 × 10^3^/well in a 96-well plate and 350 × 10^3^/well in a 24-well plate. HeLa cells at 15 × 10^3^/well in a 96-well plate; HEK293 cells at 1.5 × 10^3^/well in a 96-well plate; SH-SY5Y and PC12 at 20 × 10^3^/well in a 96-well plate.

Cells were fixed with a 2.5% glutaraldehyde solution to a final concentration of 0.5%, and incubated for 10 min. The plate was washed, dried for 1 hour, or overnight at 50°C, and stained with 100μl of methylene blue solution (filtered 1% Methylene blue dissolved in 0.1M boric acid, pH 8.5) for 1h, washed, and dried for 1h at 50°C, or overnight. Then, 200μl 0.1MHCl was added for 1h, at 37°C and read in a Plate Reader Synergy H1 at 630nm.

### 2.4 667 cell proliferation assay

Cell proliferation was measured using the Cell Titer Glo reagent (Promega) according to the manufacturer’s instructions. Briefly, 10,000 cells were plated in a white-bottom 96-well plate. Cells were treated with vehicle (DMSO), Auf (Medchem Express, HY-B1123), ACR (Sigma, A9099), and/or BSO (Medchem Express, HY-106376) at the indicated concentrations for 72 hours. Cells were incubated with Cell Titer Glo reagent for 10 min on a rocking platform, and luminescence was measured on a Synergy Neo 2 plate reader (BioTek Instruments).

### 2.5 Measurement of hydrogen peroxide in 667 cells

Hydrogen peroxide was measured using the roGFP2 and mito-GFP2 fluorescent reporter systems. The reduction/oxidation GFP sensor (roGFP2) exhibits green fluorescence in the reduced form and blue fluorescence in the oxidized form. Changes in redox state are expressed as a ratio of mean blue fluorescence to mean green fluorescence (oxidized:reduced). 667 cells expressing either a cytoplasmic roGFP2 (pLenti6.2_mCherry_roGFP2-Orp1, Addgene 155043) or mitochondria-localized roGFP2 (mito-roGFP2) (pLenti6.2_mCherry_mito_roGFP2-Orp1, Addgene155042) were plated in a 96-well cell culture plate at 100,000 cells/well. Cells were subsequently treated with vehicle (DMSO) or with Auf (0.25 μM), BSO (50 μM), and/or ACR (0.1 mM). mito-roGFP2 and roGFP2 fluorescence were excited at 405 nm and 485 nm lasers, and emission was monitored at 510 nm on an LSR Fortessa flow cytometer (BD Biosciences) and analyzed using FlowJo.

### 2.6 Western blot and molecular signaling analyses

Twenty-four hours after plating, cells were exposed to the different combinations of ACR, Auf, or BSO for a 3 hours, lysed using 0.1 ml lysis buffer (150mM Tris, pH 6.8, 10% glycerol, 2% SDS, Bromophenol Blue ,and 7ml β-mercaptoethanol/ml). Proteins were separated by 10% SDS-PAGE. Lysates were heated at 100°C for 5 min. For western blot analysis, 20–40μg of lysate was loaded on a 10% SDS-PAGE gel, and proteins were separated by electrophoresis and transferred to a nitrocellulose membrane (Whatman, Maidstone). After exposure to the corresponding primary antibody and HRP-linked second antibody, the blots were visualized by enhanced chemiluminescence and detected with Gelimager Fusion FX, (Vilber Lumart). Densitometry of immunoblots was performed with NIH ImageJ 1.53f51.

### 2.7 Preparation of GBM organoids

GBM organoids were established as previously described (Abdullah et al., 2022). Briefly, tumor tissue was obtained directy from a patient with newly diagnosed GBM undergoing resection in the operating room. The collection and use of patient-derived tissue specimens were approved by the UT Southwestern Medical Center Institutional Review Board (IRB protocol STU022011–070). All procedures involving human participants were conducted in accordance with the Declaration of Helsinki and institutional guidelines. Written informed consent was obtained from all participants prior to tissue collection.

Tumor pieces were incubated in red blood cell lysis buffer (Thermo Fisher, 00433357) at room temperature for 10 min with rocking. Tumor pieces were then incubated in organoid media containing Amphotericin B (final concentration 0.25 mg/mL). Organoid Media contained 250 mL DMEM:F12 medium (ThermoFisher, 1132033), 250 mL Neurobasal medium (ThermoFisher, 21103049), 5 mL 100X Glutamax (final conc. = 2 mM, ThermoFisher, 35050061), 5 mL MEM non-essential amino acids solution, ThermoFisher, 11140050), 5 mL HEPES (ThermoFisher, 15630080), 5 mL penicillin/streptomycin (final conc. = 100 U/mL and 100 μg/mL, respectively, ThermoFisher, 15140122), 10 mL B-27 Supplement without Vitamin A (ThermoFisher, 12587010), 5 mL N-2 Supplement (ThermoFisher,17502048), and 125 μl human insulin (final conc. = 2.375–2.875 μg/mL, Sigma Aldrich, I9278). Tissues were cut into ~1–2 mm pieces using sterile scalpels and incubated in organoid media in 24-well plates and rotated at 120 rpm in a humidified chamber at 37^∘^C, 5% CO_2_, and 21% O_2_. All methods were performed in accordance with the relevant guidelines and regulations set forth by the University of Texas Southwestern Medical Center Institutional Review Board.

### 2.8 Treatment and immunofluorescent analysis of GBM organoids

GBM organoids were treated with vehicle or AuF (1 μM), BSO (100 μM), and ACR (100 μM) for 10 days. After the treatment period finished, organoids were fixed in 4% paraformaldehyde for 15 minutes at 4^∘^C. Then, organoids were washed in PBS for 3 minutes before being incubated in 1 part SHIELD Epoxy and 7 parts SHIELD ON (both from LifeCanvas Technologies, SH-250) overnight at 4^∘^C. The next day, organoids were moved to room temperature for 3 hours. Organoids were transferred to delipidation buffer (LifeCanvas Technologies, SH-250) at 45^∘^C and incubated overnight. Organoids were then incubated in primary antibodies (1:500 Ki-67, Cell Signal, 9449S, RRID:AB_2797703; 1:500 Cleaved Caspase 3, Cell Signal 9664, RRID:AB_2070042) in PBS with 0.1% Triton X-100 at 37^∘^C for 3 days. Organoids were washed in PBS 3 times for 5 minutes each and then incubated in secondary antibodies (1:1000 Alexa Fluor 488 donkey anti-rabbit, Invitrogen, A2106, 1:1000 Alexa Fluor 568 goat anti-mouse, Invitrogen, A11004), NucSpot 680/700 (diluted as per manufacturer’s instructions, Biotium, CHM11L295), and Hoechst stain (final concentration 1.6 μM, Thermo Fisher, 33342) at 37^∘^C for 3 days. Then, organoids were washed in PBS 3 times for 5 minutes each and were index matched in 50% EasyIndex, 50% distilled water at 37^∘^C for 3 hours. Organoids were then transferred to 100% EasyIndex at 37^∘^C for 3 days. Cleared organoids were embedded in a mixture of 2% (w/v) ultra-low melting point agarose (Sigma A2576) with EasyIndex using standard sample holders (LifeCanvas Technologies). The agarose mixture was stored for a minimum of 1 day at room temperature for full hydration. A sufficient volume (~3ml per block) of hydrated agarose mixture was heated to 90 degrees C at least 3 hours before embedding. A UV penlight was used to locate the cleared organoids stored in Easyindex, which were placed into blocks according to treatment condition. Embedded blocks were stored submerged in 100% Easyindex in well-sealed containers overnight prior to imaging. Images were acquired on the LifeCanvas SmartSPIM lightsheet microscope. A 3.6x objective was used to generate images with 1.8 μm isotropic pixel resolution in X, Y and Z dimensions. Four fluorescent channels were acquired for each organoid at the indicated excitation wavelengths: 445 nm (autofluorescence), 488 nm (Cleaved Caspase 3), 561 nm (Ki-67), and 640 nm (NucSpot 680/700). Laser intensity for all channels was tuned to 100% with the exception of 640 nm, which was tuned to 70%. Custom software tools for image de-striping, contrast adjustment, stitching, and conversion of the 3D volume to Imaris format were used (LifeCanvas Technologies). Sample embedding and lightsheet microscopy were performed in the UT Southwestern Whole Brain Microscopy Facility (WBMF; RRID:SCR_017949).

All methods were performed in accordance with the relevant guidelines and regulations set forth by the University of Texas Southwestern Medical Center Institutional Review Board and by the Hebrew University Review board. This study was approved by the University of Texas Southwestern Medical Center Institutional Review Board (STU022011–070).

### 2.9 Statistical analysis

All values were presented as mean ± SEM. Statistical analysis was performed using GraphPad Prism and Microsoft Excel software. Differences among means were analyzed using two tail type t-test.

## 3. Results

### 3.1 ACR, Auf, and BSO exert cytotoxicity in U87MGMG cells

**Figure 1:**
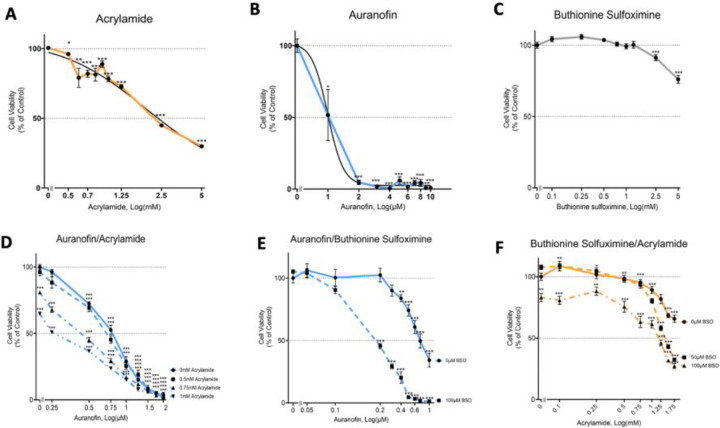
Effect of oxidizing reagents on the GBM U87MGMviability Dose-dependent cytotoxic response in U87MG cells induced by (**A**) acrylamide (ACR) (**B**) auranofin (Auf) and (**C**) buthionine sulfoximine (BSO) on their own, or by co-treatment of (**D**) Auf and ACR (**E**) Auf and BSO (**F**) BSO and ACR. Cell viability was monitored using methylene blue after 72h and represented as the mean of six replicates ±SEM. (N=8) The IC_50_ values were obtained from the nonlinear fitting of [Compound] x Cell Viability (%). Statistical significance: t-test from 0 μM control: *p value < 0.05; **p value < 0.01; ***p value < 0.005.

Initially, we assessed the cytotoxicity of ACR, Auf, and BSO on the isogenic GBMcell line U87MG. Cells were grown in a 96-well plate and treated with increasing concentrations of either ACR, Auf, or BSO, as indicated, for 72 hours, and cell viability was determined using an MTT assay (Fig. 1A,B,C). ACR inhibited cell viability in a dose-dependent manner, displaying a half-maximal inhibitory concentration (IC50) of 2.5 mM (Fig. 1A). Auf, which is known to induce ROS-dependent cytotoxicity within a micromolar range, significantly decreased cell viability, displaying an IC50 of 1 μM with complete cell death at 2 μM (Fig. 1B). In contrast, incubation of the cells with increasing concentrations of BSO showed only a small effect of ~ 25% reduction at 5 mM and minimal effect at 1.25 mM on U87MG viability (Fig. 1C).

### 3.2 The two-reagent combinations ACR/AuF, AuF/BSO, and BSO/ACR exert synergistic cytotoxicity in U87MG cells

Next, we assessed the cytotoxicity of two-reagent combinations: ACR/Auf, Auf/BSO, and BSO/ACR in U87MG cells. The cytotoxic effect of Auf in combination with increasing concentrations of ACR up to 1 mM, was tested at concentrations ranging from 0.1 to 2 μM (Fig. 1D). At 0.25 μM, Auf by itself had no effect on cell survival. We found that cytotoxicity mediated by 0.25 μM Auf was increased approximately 25% by 0.75 mM ACR, and approximately 50% by 1 mM ACR. No synergistic cytotoxicity was observed at 0.5mM ACR. Cytotoxic synergy was observed throughout the Auf concentration range.

Next, we tested the cytotoxic effect of Auf at increasing concentrations up to 1 μM, in combination with 100μM BSO (Fig. 1E). In the presence of 100μM of BSO, there was a strong shift in the Auf death curve to lower concentrations, which indicates potentiation of Auf’s cytotoxic effects and was consistent with strong synergy between these two reagents, previously shown in vivo and in several cellular systems (Simons et al., 2009), (Sobhakumari et al., 2012), (Roh et al., 2017), (Fath et al., 2011), (Kiebala et al., 2015), (Li et al., 2015), (You and Park, 2016), (Haβ et al., 2016), rhabdomyosarcoma (Habermann et al., 2017), (Rashmi et al., 2018), (Fazzari et al., 2022), (Cui et al., 2022), (Jamali et al., 2024).

Lastly, we tested the effect of ACR and BSO on cell survival. Increasing concentrations of ACR up to 1.75 mM, were tested in the presence of 50 μM and 100 μM BSO (Fig. 1F). While no significant shift was observed in the ACR-survival curve with 50 μM of BSO, a slight shift was noticed with 100 μM BSO, displaying IC_50_ ~1.25 mM.

### 3.3 ACR/AuF/BSO exerts synergistic cytotoxicity in U87MGMG cells

**Figure 2:**
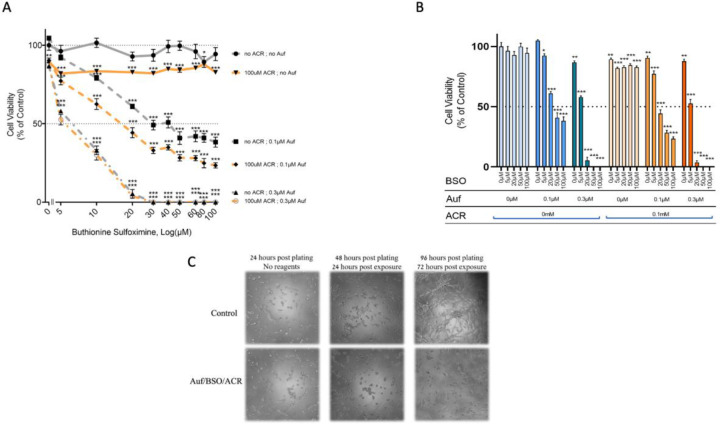
Synergistic lethality and the effect on morphology by the ACR/Auf/BSO dose combination on U87MG cells **(A**) Dose-dependent cytotoxic response in U87MG cells exposed to triple combination ACR/Auf/BSO at different combinations as indicated. A dose-dependent response of U87MG cells and (**B**) highlighted as bar graph. The IC_50_ values were obtained from the nonlinear fitting of [Compound] x Cell Viability (%). Statistical significance mean of six replicates ±SEM (n=8), t-test *p value < 0.05; **p value < 0.01; ***p value < 0.005 (**C**) Effect on morphology in U87MG cells after 72 hours in control cells and after exposure to 100μM ACR/0.3μM Auf/20μM BSO, at 24h post plating and immediately prior to reagent application, 48h post-plating and 24h post reagent application, and 96h post plating and 72h post reagent application.

Since 100μM BSO displayed no effect on cell survival and showed no synergy with 100μM ACR, we tested the three-reagent combination by increasing concentrations of BSO(up to 100μM with or without 100 μM ACR, in the presence of 0.1 or 0.3 μM Auf (Fig. 2A,B). As shown, strong synergy was observed with BSO and Auf as previously reported. A slightly higher efficacy was observed by the addition of ACR, particularly in the presence of 0.1μM Auf. We should note that since the ACR electrophile covalently binds to the highly nucleophilic cysteine thiol, its effect could become more prominent under longer assay conditions or within *in vivo* experiments. Based on these survival data, we selected a combination of the three reagents BSO (20μM)/Auf (0.3 μM)/ACR (100μM) to monitor morphological changes in U87MG cells. As shown in Fig. 2C, 24 hours post-exposure, the cells treated with the three-reagent combination exhibited a compressed, rounded morphology, indicative of cellular stress and apoptotic changes. In contrast, the control cells continued to proliferate and began to branch out, forming connections (Fig. 2C). Seventy-two hours post-exposure, the treated cells were sparse, with many remaining in a rounded, compressed state. The control cells, however, reached confluence and developed large astrocyte-like structures, suggesting normal growth and differentiation. These morphological observations further support a significant cytotoxic effects on GBM cells.

### 3.4 Synergistic lethality and ROS production in IDHwt GBM spheres

**Figure 3:**
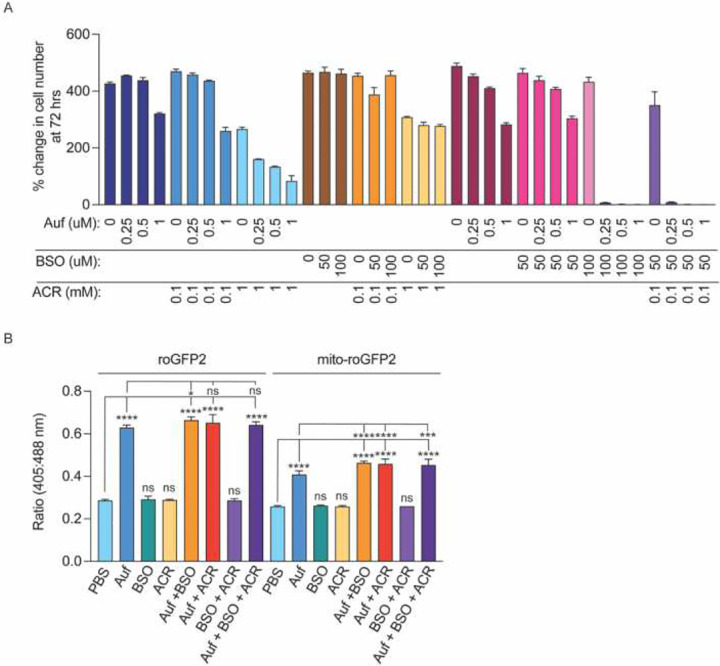
Synergistic lethality and hydrogen peroxide measurements in the IDH wt 667 GBM spheroid cells Dose-dependent cytotoxic response in IDH wt 667 GBM cells induced by thiol-reactive reagents ACR, Auf and BSO on their own alone, or by co-treatment of two or three reagents combined. (%). Statistical significance mean of six replicates ±SEM (n=8), t-test *p value <0.05; **p value < 0.01; ***p value < 0.005. Hydrogen peroxide was measured using the roGFP2 and mito-GFP2 fluorescent reporter systems in 667 GBM cells expressing either mCherry-roGFP2 or mCherry-mito-roGFP2. The cell were incubated with PBS, 250 nM Auf, 50 μM BSO, and/or 100 μM ACR either alone or in combination. ROS production was reported as the ratio of blue:green fluorescence at 405:488 nm excitation, respectively. ***p value < 0.005 **** p value < 0.001

We next assessed the viability of isocitrate dehydrogenase (IDH) wild-type (wt) patient-derived primary GBMs with different combinations of ACR, BSO and Auf. Similar to U87MG cells, survival of GBM tumor spheres (667) was strongly affected by the Auf/BSO and the ACR/BSO/Auf combinations (Fig. 3A). Treatment revealed a higher sensitivity of the 667 cells to the combination in comparison to U87MG cells. In the presence of 0.1mM ACR/50μM BSO, cell death was exhibited at 0.25μM Auf, which was one-half the concentration required if ACR was absent. Hence, the addition of ACR appeared to further enhance oxidative stress-induced cytotoxicity. We also assessed the production of ROS using the genetically encoded fluorescent ROS reporter roGFP and a mitochondrially targeted roGFP, called mito-roGFP. Treatment with Auf led to high levels of total cellular ROS and mitochondrial ROS (Fig. 3B). BSO significantly elevated mitochondrial ROS more than Auf alone, though adding ACR did not elevate mitochondrial ROS further. These data indicate that Auf and BSO induce significant ROS in patient-derived GBM cells, which likely induce cytotoxicity.

### 3.5 ACR/Auf/BSO exert synergistic toxicity in A431 epidermoid carcinoma cells

**Figure 4:**
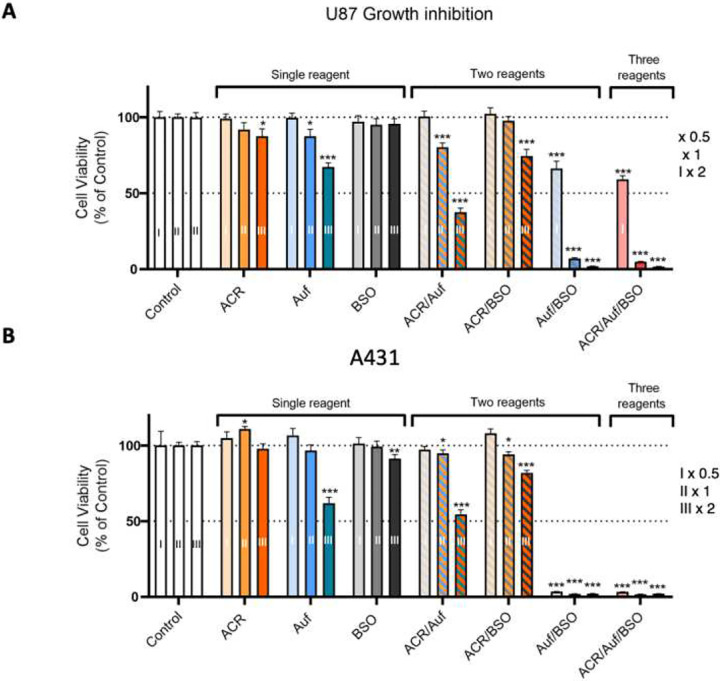
Synergistic lethality induced by ACR/Auf/BSO in U87MG, and A431 epidermal carcinoma cells Survival of (**A**) U87MG cells and (**B**) A431 cells 72 h after incubation with one, two or three reagents at concentrations of one half- the combination concentration 50μM ACR/0.15 μM Auf/10 μM BSO (I,) the full-dose combination concentration 100μM ACR/0.3 μM Auf/20 μM BSO (II) and twice the dose combination concentration 200μM ACR/Auf 0.6 μM/BSO 40 μM. Each data point shows the mean ± SEM (n=8), t-test *p value < 0.05; **p value <0.01; ***p value < 0.005.

The potential synergistic lethality of the combination ACR/Auf/BSO (0.1 mM/0.3 μM/20 μM), as revealed in U87MG cells (Fig.4A), was evaluated by comparing the survival rates of U87MG and A431 epidermal carcinoma cells over 72 hours. A431, a human epidermoid carcinoma cell line originally derived from a skin biopsy of a patient with vulvar squamous cell carcinoma, is often used as a model cell system to test the effects of anti-tumor drugs. We tested a half-dose combination ACR/Auf/BSO (0.05 mM/0.15 μM/10μM) [column (I)], full-dose combination ACR/Auf/BSO (0.1 mM/0.3 μM/20μM [column (II)], and a double-dose combination ACR/Auf/BSO (0.2 mM/0.6 μM/40 μM [column (III)] (Fig. 4B). By themselves, each one of the three reagents demonstrated a similar effect in both U87MGMG and A431 cells (I). A similar effect was seen in the ACR/Auf combination (orange/blue stripes) in both single and double doses as well as the ACR/BSO double dose. The double-dose of Auf (0.6 μM) and BSO (40 μM) had a slight stronger effect (8%) in A431 relative to U87MG cells. A similar effect was seen in cells treated with ACR/Auf (orange/blue stripes), both with the single and the double doses. The A431 population was completely abolished by Auf/BSO and the 3-reagent combination, even at half the dose, as opposed to a mere 40% decrease in the U87MG population at the same dose.

### 3.6 Molecular signaling associated with ACR, Auf and BSO

Next, we explored potential molecular mechanisms responsible for the lethality induced by the ACR/Auf/BSO combination in A431 cells. These cells, which express very high levels of EGFR, are commonly used in studies of cancer-associated signaling pathways.

**Figure 5:**
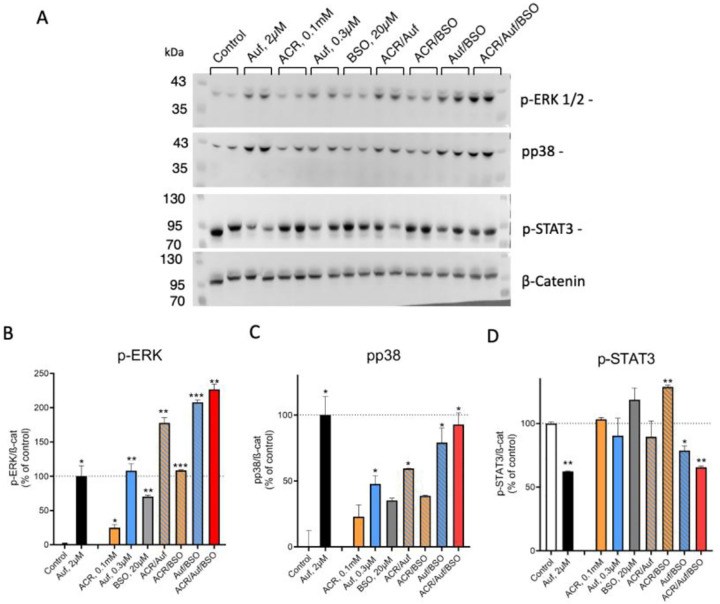
The effects of ACR/Auf/BSO on phosphorylation of ERK1/2 p38 and STAT3 in A431 cells **(A**) Induction of ERK1/2 phosphorylation in A431 cells exposed for 3h to single, double, and triple concentrations of ACR, Auf, and BSO, as indicated. Values were obtained from cell lysates in a western blot analysis using the corresponding anti-pERK1/2, p38MAPK and pSTAT3 antibodies. Quantification of (**B**) pERK1/2 phosphorylation (**C**) p38MAPK phosphorylation and (**D**) pSTAT3 phosphorylation, using 2μM Auf as a positive control. Each data point shows the mean ± SEM (n=2), t-test *p value < 0.05; **p value < 0.01; ***p value < 0.005.

The single, double, and the triple reagent combinations ACR/Auf/BSO (0.1 mM/0.3 μM/20 μM) were applied to the cells for 3 hours, and the effect on the signaling pathways was examined by monitoring the phosphorylation of ERK1/2, p38, and STAT3. We used 2μM Auf, which is known to strongly activate ERK1/2 and p38 phosphorylation. As shown in Fig. 5A, there was a strong increase in ERK1/2 phosphorylation by Auf/BSO and ACR/Auf/BSO compared to the individual application of the reagents. A significant increase was also observed in the phosphorylation of p38MAPK. Under all conditions, there was a smaller effect on ERK1/2 phosphorylation compared to the high Auf positive control. When combined, ACR/Auf showed similar results to Auf alone (~11%), while ACR/BSO showed a significant increase compared to BSO alone (~30%). Auf/BSO demonstrated a further increase relative to ACR/Auf (~22%), and the triple combination (~35%) further increased the phospho-protein level to a level similar to the high Auf condition. The strong elevation of phosphorylated p38 indicates activation of the p38 inflammatory/apoptotic pathway (Fig. 5A,C). The transcription factor STAT3, which plays a major role in cancer, is highly expressed in human GBM tissues, and rarely expressed in normal brain tissues (Li et al., 2010). We used as a control 2 μM Auf as a control, which displayed 38% reduction in pSTAT3 level. A smaller decrease was observed using 0.3 μM Auf or 0.1mM ACR, with an increase in 20μM BSO. Also, the combination of ACR/BSO showed an increase in the pSTAT3 level. A prominent decrease in pSTAT3 expression was observed by incubation of A431 cells with Auf/BSO and with Auf/BSO/ACR (Fig. 5A,D). This decrease in pSTAT3 level can be attributed to the irreversible inhibition of thioredoxin reductase by Auf (Busker et al., 2020).

### 3.7 The effect of ACR/Auf/BSO on survival of neuronal primary cells

**Figure 6:**
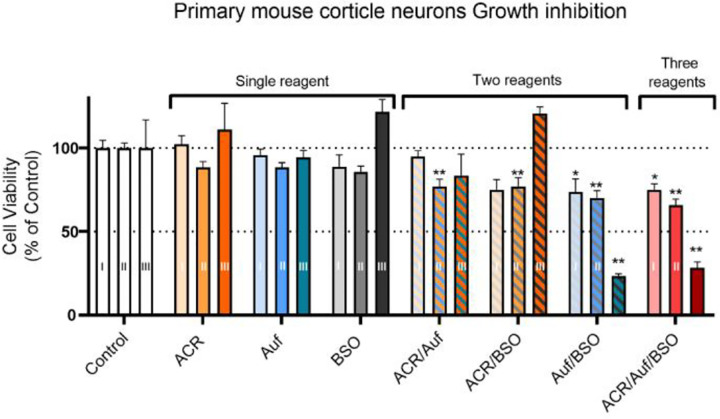
Growth inhibition in primary mouse cortical neuronal cells Growth of primary mouse neuronal cells grown for two weeks and exposed for 72 h cells to one, two or three reagents ACR, Auf, and BSO. Concentrations were of (I) one half the combination concentration 50 μM ACR, 0.15 μM Auf, 10 μM BSO, (II) the combination concentration 100 μM ACR, 0.3 μM Auf, 20μM BSO and (III) twice the combination concentration 200 μM ACR, 0.6 μM Auf, 40 μM BSO. Each data point shows the mean ± SEM (n=2); t-test *p value < 0.05; **p value < 0.01.

Next, we investigated the effects of ACR, Auf, BSO, and their combinations on cell survival in non-cancerous primary mouse neuronal cultures isolated from mouse embryonic cortex (E18–19) cells. Cortical cells were extracted from mouse embryos and assessed for survival (Fig. 6). In the 72-hour survival assay, ACR, Auf, and BSO individually showed no significant impact on primary cell survival at half- (I), full- (II), or double- (III) dose concentrations. However, when two reagents were combined, a reduction in the cell population of approximately 25% was observed at all concentration levels in the ACR/BSO and ACR/Auf combinations. The double dose of Auf/BSO (III) resulted in a ~75% decrease in cell survival, similar to the established full-dose combination (II) of ACR/Auf/BSO, which showed a similar reduction to the two-reagent combination at half dose (I) (~25%), a slightly larger decrease at the full-dose dose (II) (~35%), and a pronounced decrease (~70%) at the double dose (III).

### 3.8 Primary cortical cells do not exhibit significant differences in molecular signaling in response to ACR, Auf, and BSO

**Figure 7:**
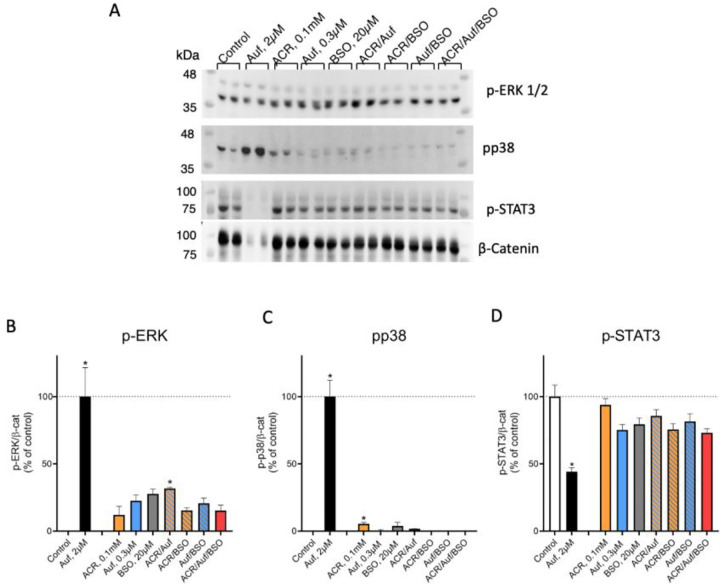
The effects of ACR/Auf/BSO and combinations on phosphorylation of ERK1/2, p38MAPK, and STAT3 in primary mouse cortical neuronal cells **(A**) Induction of ERK1/2 phosphorylation in A431 cells exposed for 3h to single, double, and triple concentrations of ACR, Auf, and BSO, as indicated. Values were obtained from cell lysates in a western blot analysis using the corresponding anti-pERK1/2, p38 and pSTAT3 antibodies. Quantification of (**B**) pERK1/2 phosphorylation, (**C**) p38 phosphorylation, and (**D**) pSTAT3 phosphorylation. 2μM Auf was used as a positive control. Each data point shows the mean ± SEM (n=2), t-test *p value < 0.05; **p value < 0.01;

Next, we examined the effects of single, double, and triple combinations of ACR, Auf, and BSO on the activation of ERK1/2, p38, and STAT3 signaling pathways in primary cortical cells. As shown in Fig. 7, cells were treated with the reagents at half (I), full (II), and double doses (III) for 3 hours. Activation of the signaling pathways was assessed by monitoring the phosphorylation of ERK1/2 (Fig. 7A), and p38MAPK (Fig. 7B), and STAT3 (Fig. 7C). As shown, pERK1/2 activation increased slightly, by up to 20%, following exposure to various combinations, with the highest increase observed in the ACR/Auf condition (Fig. 7A). In contrast, p38 and STAT3 showed no significant activation under any of the tested conditions. These findings suggest that primary cortical cells exhibit resistance to the toxicity of these reagents compared to A481 cells.

### 3.9 Synergistic lethality by ACR, Auf, and BSO in U87MGΔEGFR (U87MGΔ) cells

U87MGΔEGFR GBM cells are known to display constitutively active EGFR due to an in-frame deletion of exons 2 to 7 in the ligand binding domain of the EGFR.

**Figure 8:**
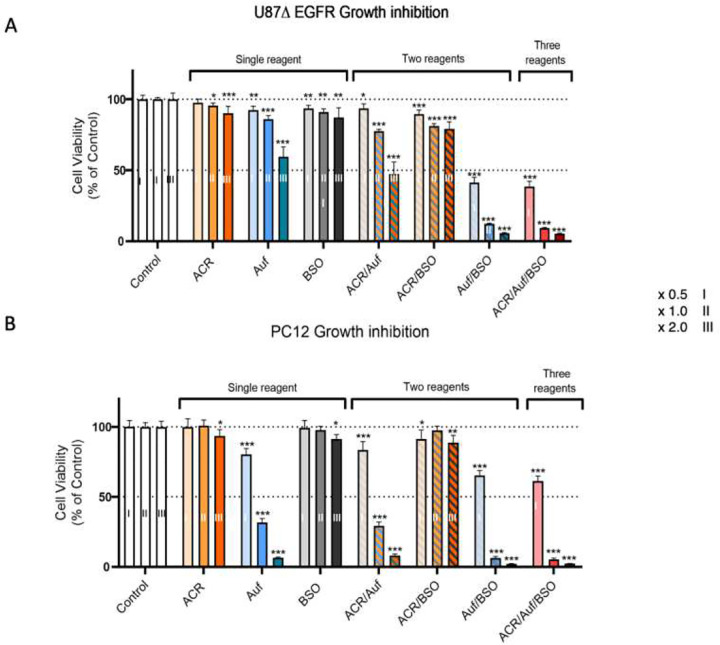
Synergistic lethality induced by ACR/Auf/BSO in U87MGΔEGFR, and PC12 cells Survival of (**A**) U87MGΔEGFR cells and (**B**) PC12 cells 72 h after incubation with single, double, and triple reagent at concentrations of (I) one half the combination concentration 50 μM ACR/0.15 μM Auf/10 μM BSO (II), the combination concentration 100 μM ACR/0.3 μM Auf/20 μM BSO, and (III) twice the combination concentration 200 μM ACR/Auf 0.6 μM/BSO 40 μM.Each data point shows the mean ± SEM (n=8), t-test *p value < 0.05; **p value < 0.01; ***p value < 0.005.

These cells exhibit various mechanisms of drug resistance, including enhanced production of GSH, oxidative stress responses, ferroptosis, heat shock protein expression, and apoptosis-related gene activities. To evaluate the synergistic lethality of our selected triple combination (ACR/Auf/BSO at 0.1 mM/0.3 μM/20 μM), we treated U87MGΔEGFR cells with one-half (I), full (II), and double (III) dose. The synergistic lethality was determined 72 hours after exposure to the combinations (Fig. 8A).

We found that ACR and BSO at half- (I), full- (II), or double- doses (III), had minimal impact on cell survival. In contrast, Auf alone induced significant cell death (~40%) at the double dose (III). The most pronounced effect was observed with the Auf/BSO combination, which demonstrated synergistic lethality, significantly enhancing cytotoxicity compared to either reagent alone. This effect was evident at half-dose (I) and exceeded 90% at the full- (II) and double (III) dose. There was no difference between the effects of Auf/BSO and ACR/Auf/BSO/ at the three different combination doses. A431 cells (Fig. 4B), showed a complete eradication by Auf/BSO and ACR/Auf/BSO combination at (I), (II), and (III).

### 3.10 Synergistic lethality induced by ACR, Auf, and BSO in SH-SY5Y and PC12 cells

Next, we explored ACR, Auf, and BSO-induced PC12 and SH-SY5Y cell lethality. PC12 cells are a cell line derived from a pheochromocytoma of the rat adrenal medulla and have been extensively characterized for catecholamine secretion triggered during membrane depolarization mediated by voltage-gated calcium channels and neurotransmitter receptors. SH-SY5Y cells are a cloned subline of a neuroblastoma cell line from a metastatic bone tumor and are often used as *in vitro* models of neuronal function and differentiation. Synergistic lethality in these cell lines was studied using the same protocol (see above). We applied our chosen triple combination dose of ACR/Auf/BSO: 0.1mM/0.3μM/50μM, at one-half dose (I), a full-dose (II), and double-dose (III) to PC12 (Fig. 8B) and to SH-SY5Y cells. Cell viability was examined 72 h after exposure. In PC12 cells treated with a single reagent ACR, or BSO alone or with two reagents, no effect on cell viability was observed at I, II and III) doses, while a strong effect was observed with Auf alone (II and III), ACR/Auf, and Auf/BSO (II, and III). Further reduction in cell viability was observed with the three reagents at all three doses. Similar effects were observed in SH-SY5Y cells (**Fig. S1**).

### 3.11 Auf, BSO, and ACR reduce proliferation of GBM tissue organoids

**Figure 9:**
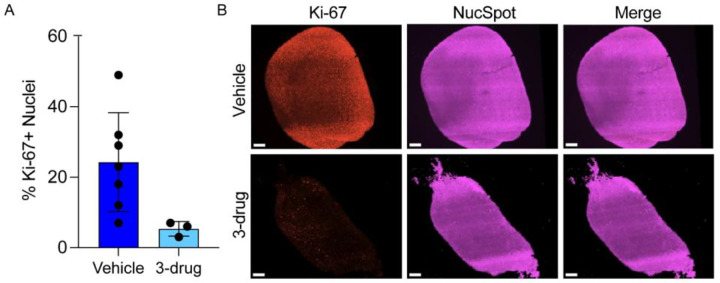
Auf, BSO, and ACR reduce proliferation of human GBM tissue organoids GBM2603 tissue organoids were treated with vehicle or 1 μM Auf, 100 μM BSO, and 100 μM ACR for 10 days. Tissue organoids were stained for Ki67, cleared, and then imaged through light sheet microscopy. (**A**) Nuclei were stained with NucSpot, and % positive nuclear staining was calculated in Imaris (**B**) Representative immunostaining of Ki67+ nuclei (red) and NucSpot stain (magenta). p-value shows the comparison to Vehicle.

To assess the effect of Auf, BSO, and ACR on tumor proliferation in a relevant human model, we developed a GBM tissue organoid model, where 1–2 mm pieces of fresh resected GBM patient tissue are incubated in culture media and treated with drugs for up to 10 days. We incubated tissue organoids from a patient with newly diagnosed GBM with 1 μM Auf, 100 μM BSO, and 100 μM ACR for 10 days and then fixed and stained the organoids for the proliferation marker, Ki-67, before tissue clearing and imaging using light sheet microscopy. We found that Ki-67 expression was significantly reduced in organoids treated with the 3-drug combination versus vehicle (Fig. 9A, 9B), indicating that these drugs also reduce growth in fresh GBM tissue

## Discussion

Our study reveals, for the first time, cell-induced toxicity through a three-drug combination strategy involving Auf, BSO, and ACR. This combination induces synergistic lethality in three GBM cell lines, (U87MG, U87MGΔEGFR, 667 GBM spheroids), A431, PC12, SH-SY5Y, and HELA cells by targeting thiol groups and disrupting multiple redox-related cellular pathways. Moreover, a ten-fold difference in proliferation was observed between vehicle and 3-drug groups in patient-derived GBM organoids. This strategy offers a promising novel approach for enhancing cancer therapy and potentially reducing cancer cell resistance to treatment.

### Lethality synergy induced by three different thiol-reactive reagents

Cancer cells display higher susceptibility to cellular insults compared to non-cancerous cells. This vulnerability makes combination therapy, which simultaneously targets multiple apoptotic pathways, an effective strategy widely employed in cancer treatment. Synergistic toxicity in various cancer cells has previously been demonstrated through the dual targeting of the thioredoxin (TrxR/Trx1) and GSH (GSH-GSSG) redox systems using Auf and BSO (Simons et al., 2009), (Fath et al., 2011), (Sobhakumari et al., 2012), (Roh et al., 2017), (Martinez-Jaramillo et al., 2024), (Jamil 2024) and others. Here, we demonstrate that the addition of ACR, thiol-reactive molecule that covalently modifies redox-related proteins and disrupts critical signaling pathways, further amplifies the cytotoxic effects of Auf and BSO. This triple-reagent combination leads to enhanced suppression of cell growth in various cancer cell lines, including U87MG, U87MGΔEGFR, A431, SH-SY5Y, and PC12 cells.

ACR promotes oxidative stress mainly via Michael’s addition by alkylation of the nucleophilic sulfhydryl groups in proteins associated with the redox state of the cell (e.g. GSH, thioredoxin, N-ethylmaleimide sensitive factor (NSF), SNAP-25, adenosine deaminase and others) (Schwend et al., 2009), (Nair et al., 2014). The design of small molecule drugs with ACR covalent warheads, like the tyrosine kinase inhibitors (Liang et al., 2024), suggest that ACR might increase lethality not only due to the alkylation of GSH and thioredoxin, but also through alkylation of a variety of Cys residues.

We established a minimal lethal combination of ACR (0.1 mM), Auf (0.2 μM), and BSO (20 μM), applied over a 72-hour incubation period. This minimal drug combination exhibited a remarkable synergistic effect in inducing cell lethality, as none of the individual reagents demonstrated toxicity at these concentrations when applied separately. The addition of ACR as a third reagent, which resulted in complete cell death, enabled the use of lower concentrations of BSO and Auf to achieve this effect compared to the combination without ACR. For example, in IDH wild-type GBM spheroids (667 cells), the synergistic combination of Auf, BSO, and ACR required only half the concentration of Auf (0.15μM) to achieve cell death compared to the Auf/BSO combination without ACR. These same concentrations induced ROS production in patient-derived GBM cells.

These results suggest an increased cellular sensitivity to the three-reagent combination, highlighting the effectiveness of incorporating ACR to form a triple-drug treatment. Given the significant synergistic effects observed with Auf and BSO in clinical settings, the incorporation of ACR introduces an additional disruptive cellular pathway that could potentially slow down the rapid mutational resilience of cancer cells in vivo. This approach may enhance the therapeutic efficacy against GBM, which is known to exhibit a unique susceptibility to disruptions in Cys-containing proteins (Noch et al., 2024).

A431 cells were the most sensitive, showing complete growth inhibition even with Auf/BSO at half the concentration [(Auf 0.15μM/BSO 50μM; (I)]. U87MG, U87MGΔEGFR, and PC12 cells were inhibited by Auf/BSO at the full-dose concentration (II), while complete lethality was observed in all cancer cell lines (U87, A431, PC12, and 667 cells) incubated with double dose concentration (III).

In contrast, in primary neocortical cells, cytotoxicity was significantly lower, showing (~40%) by Auf/BSO or the ACR/Auf/BSO combination dose (II). These findings indicate a preferential cytotoxic effect on cancer cells and suggest the potential therapeutic selectivity of this combination.

### Molecular pathways affected by a three-thiol reagent combination

The MAPKs and the transcription factor STAT3 are among the numerous molecular pathways that are involved in tumorigenesis and proliferation. They play a major role in proliferation, metastasis, and chemotherapy resistance of GBM and other cancer cells, and are widely explored as a potential target for anticancer therapeutics. Through their activation of inflammatory and apoptotic signaling pathways, MAPK and the STAT3 family of proteins were shown to promote uncontrolled cell division, enhanced survival, and resistance to therapy, contributing to the pathogenesis of GBM (Bromberg, 2002) (Fu et al., 2023), (Hasan et al., 2024), (Loftus et al., 2024), (Brantley et al., 2008), (Dasgupta et al., 2009).

STAT-3 activation mainly by cytokines and growth factors induces tyrosine phosphorylation and results in expression of genes that control cell proliferation, survival and differentiation (Xie et al., 2019). Aberrant activation of STAT-3 has been identified in GBM and also in a number of other human cancers (Wong et al., 2026), (Muthu et al., 2026). Both STAT3 and p-STAT3 are highly expressed in human GBM tissues, and low or rarely expressed in normal brain tissues (Li et al., 2010). In human GBM, p-STAT3 expression is present in up to 60% of cells, and is closely related to invasion and metastasis, the histological grade, and is associated with poor prognosis (Lindemann et al., 2011).

We demonstrate that the increase in ERK1/2 and p38MAPK phosphorylation, consistent with previous studies highlighting strong Auf- and ACR-induced MAPK activation (Bachnoff et al., 2011) (Cohen-Kutner et al., 2013), (Martin et al., 2024), together with the inhibition of pSTAT3 in A431 cells mediated by the three-thiol reagent combination (ACR, Auf, and BSO), correlates with the observed synergistic lethality. These findings suggest that the coordinated modulation of these signaling pathways contributes to the enhanced cytotoxic effects of combination treatment.

The increase in ERK1/2 and p38 phosphorylation induced by the ACR/Auf/BSO combination was comparable to the levels observed with the Auf/BSO treatment. However, the ACR/Auf/BSO combination demonstrated greater efficacy in reducing pSTAT3 levels compared to Auf/BSO alone. This enhanced reduction in pSTAT3 may contribute to the superior cytotoxicity observed with the three-reagent combination. The decrease in STAT3 by ACR/Auf/BSO or by Auf/BSO is consistent with Auf’s ability to inhibit TrxR1 (Busker et al., 2020). Interestingly, Auf has been also found to target peroxiredoxin 6, which is a multifunctional antioxidant enzyme involved in ferroptosis, and thereby increases cancer cells death (Inague et al., 2024).

In primary cells, the three-thiol reagents at the combination concentrations, whether used individually or in combination, had no effect on p38 or pSTAT3. Only a minimal effect (<30%) on ERK1/2 phosphorylation was observed with the Auf/BSO combination. These results correlate with the absence of cell toxicity associated with these thiol reagents in primary cells, further highlighting the selective toxicity of the triple-agent combination in cancer cells.

A more advanced approach for determining drug efficacy was achieved through the use of three-dimensional organoids. Using organoids derived from cancer patients as ex vivo disease models, which preserve tumor heterogeneity and simulate the brain microenvironment, we observed an approximately 10-fold difference in proliferation between the vehicle-treated group (30%) and the three-drug-treated group (3%). These models can be used to predict patients’ therapeutic responses to various antitumor drug combinations within just 1–2 weeks. Using patient-derived organoids, these results further support the therapeutic potential of a combination of three oxidizing agents targeting different molecular pathways.

The high cytotoxicity observed through co-targeting TrxR/Trx1 and GSH-GSSG systems in GBM cells using the ACR/Auf/BSO combination, along with its minimal effects on primary neuronal cells, and with their ability to cross the blood-brain barrier, highlights their potential as oral anticancer drugs for GBM clinical treatment. Importantly, this 3-drug combination reduced proliferation of fresh GBM tissue organoids, validating their use in a human GBM model.

*In summary*, co-targeting GBM cells with Auf and BSO has previously demonstrated a strong synergistic lethality. Here, we introduced a third thiol-reactive reagent, ACR, which covalently modifies and inactivates thioredoxin, GSH, and additional other redox-related proteins.

We demonstrate that the combination of Auf/BSO with ACR markedly enhances synergistic cytotoxicity in U87MG, U87MGΔEGFR, A431, SH-SY5Y, and PC12 cells. This effect was associated with activation of ERK1/2, p38 MAPK, and pSTAT3 signaling pathways. Moreover, the approximately ten-fold reduction in proliferation observed in patient-derived organoids treated with the three-drug combination, relative to vehicle-treated controls, further supports the therapeutic potential of this strategy for the treatment of GBM, one of the most aggressive primary brain tumors in adults.

Importantly, primary mouse cortical neurons exhibited markedly lower toxicity and no significant activation of these signaling pathways, underscoring the potential therapeutic selectivity and safety profile of this approach for GBM treatment.

We propose that the addition of ACR, which introduces a third mechanism of redox disruption, may further impede the rapid emergence of adaptive resistance in cancer cells. This strategy therefore represents a promising therapeutic approach for improving treatment efficacy in GBM and potentially other malignancies.

## Supplementary Material

Supplementary Files

This is a list of supplementary files associated with this preprint. Click to download.

- Suppl.Info.pdf

## Figures and Tables

**Table:** Key Materials

REAGENT or RESOURCE	SOURCE	IDENTIFIER
**Antibodies**		
Rabbit monoclonal anti-pp38 MAPK (T180/Y182)	Cell Signaling	Cat#4511S
Mouse monoclonal anti-pERK1/2(pT202/pY204.22A)	Santa Cruz	Cat#Sc-136521
Rabbit anti-p-STAT (Tyr705)	Cell Signaling	Cat#9145S
Mouse monoclonal anti-β-catenin (15B8)	Cell Signaling	Cat#37447S
Anti-mouse IgG, HRP-linked	Cell Signaling	Cat#7076S
Anti-mouse IgG, HRP-linked	Cell Signaling	Cat#7074S
**Chemicals, peptides, and recombinant proteins**		
ACR for molecular biology, ≥99% (HPLC)	Sigma-Aldrich	A9099-25G; CAS: 79-06-1
Auf ≥98% (HPLC)	Sigma-Aldrich	A6733-10MG; CAS:34031-32-8
Auf	MedChemExpress,	HY-B1123
Auf ≥98% (HPLC)	Enzo	Cat#BML-EI206-0100; CAS:34031-32-8
D,L-Buthionine-(S,R)-Sulfoximine, gamma-glutamyl cystine synthase inhibitor	MedChemExpress,	HY-106376A
D,L-Buthionine-(S,R)-Sulfoximine, gamma-glutamyl cystine synthase inhibitor	Abcam	ab144943
**Software and algorithms**		
ImageJ	Wayne Rasband & Contributors, NIH, USA	http://imagej.nih.gov/ij
GraphPad Prism 8.0.2 (263)	GraphPad Software Inc.	https://www.graphpad.com/features
Microsoft Excel	Microsoft Corporation	https://www.microsoft.com/he-il/microsoft-365/excel
**Experimental models: Cell lines**		
U87MGMG10	A. Levitzki laboratory (Hebrew University, Israel)	N/A
A431	A. Levitzki laboratory (Hebrew University, Israel)	N/A
Primary neuronal culture isolated from C57Bl6 mice embryonic cortex (E18-19)	Umschweif-Nevo lab	Adi Cohen:
U87Δ MG(EGFR 2-7)	A. Levitzki Hebrew University, Israel)	N/A
SH-SY5Y	H. Soreq Hebrew University, Israel)	N/A
PC12	ATCC (Washington, DC, USA)	N/A
HeLa	A. Levitzki Hebrew University Israel)	N/A
HEK293	A. Levitzki laboratory (Hebrew University, Israel)	N/A
IDH wt 667 GBM spheroid cells	C. Brennan MSKCC NewYork, NY	N/A

## Data Availability

All original data from this manuscript will be made available upon reasonable request

## Highlights-

- A 3-drug combination of ACR, BSO, and Auf causes complete cell death in glioblastoma cells U87MG, U87MGΔEGFR.
- The synergy observed is strongly associated with increased activation of ERK1/2 and p38MAPK phosphorylation and inhibition of STAT3.
- The 3-drug combination induces whole-cell and mitochondrial reactive oxygen species (ROS) production in patient-derived GBM cells.
- The 3-drug combination reduces proliferation of a human GBM tissue organoid model.

## Abbreviations

ACR: acrylamide
Auf: auranofin
CREB: cyclic AMP response element binding protein
EGFRΔ: EGFR variant (del2-7 EGFR)
ERK1/2: extracellular signal-regulated kinase 1/2
GBM: glioblastoma
JNK1/2: c-Jun N-terminal kinase
DL-BSO: l-*S*,*R*-buthionine sulfoximine
MAPK: mitogen activated kinase
p38MAPK: p38 mitogen-activated protein kinase
STAT3: signal transducer and activator of transcription 3
Trx1: thioredoxin1
TrxR: thioredoxin reductase
TXM: thioredoxin mimetic
